# Prevalence and associated factors of sexual dysfunction in female hemodialysis patients: first report from Somalia

**DOI:** 10.1186/s12905-024-02902-w

**Published:** 2024-01-18

**Authors:** Asir Eraslan, Abdikarim Hussein Mohamed, Ahmed Muhammad Bashir, Abdulkamil Abdullahi Adani, Sertac Cimen

**Affiliations:** Mogadishu Somalia Turkish Training and Research Hospital, Mogadishu, Somalia

**Keywords:** Female sexual dysfunction, End-stage renal disease, Hemodialysis

## Abstract

**Background:**

Sexual dysfunction is frequent in female hemodialysis patients and is related to poorer quality of life. It is often a neglected topic associated with marked distress and interpersonal difficulties.

**Objective:**

Few studies are reported from Sub-Saharan African Countries (SSA) regarding female sexual dysfunction (FSD) in (HD) patients. The study aims to explore the prevalence and associated factors of FSD in female HD at a sole dialysis centre in Somalia.

**Method:**

Over a one-month period, a cross-sectional study was conducted among women with end-stage renal disease aged 18–50 years who were undergoing a dialysis program for at least three months at the dialysis center of our hospital. The participants were married, and they were living with their partners. Data regarding the sociodemographic features, clinical characteristics, frequency of sexual intercourse per week, and the Female Sexual Function Index (FSFI) scores were collected using a standard face-to-face interview questionnaire.

**Results:**

During the study period, a total of 115 participants were eligible for the study’s inclusion criteria. The mean patient age was 38.5 ± 9.3 years. The most common cause of ESRD was diabetes, which accounted for 53%, followed by hypertension (26.1%) and glomerulonephritis (9.6%). The mean duration of dialysis was 2.9 ± 1.4 years, and approximately two-thirds of the participants (62.5%) were in the program for more than three years. Regarding the frequency of sexual intercourse, 61.7% of female participants performed sexual intercourse less than once time/a week. The prevalence of FSD was 92.2% (*n* = 106) of all participants. The mean FSFI score of the participants was 16.05 ± 4.48. Longer duration of dialysis program (i.e., more than four years), increasing age (i.e., > 35 years), those with diabetes had scored lower overall FSFI scores.

**Conclusion:**

The prevalence of female sexual dysfunction among Somali female hemodialysis patients was very high, representing a significant problem in end-stage renal disease (ESRD). Our study findings revealed that increasing age, diabetes, and duration of dialysis negatively impact female sexual function and are significantly associated with FSD.

## Background

Sexual dysfunction is frequent in female hemodialysis patients and is related to poorer quality of life [1]. It is often a neglected topic associated with marked distress and interpersonal difficulties. In different age groups, 25–71% of women experience sexual dysfunction [1]. Women’s sexual dysfunction worsens with age due to endocrine-hormonal, neurological, and psychological issues, trauma, particularly gynecologic and pelvic surgical procedures, chronic illnesses like cardiovascular disease, hyperlipidemia, and diabetes, as well as a variety of medications [2].

A significant chronic condition that can lead to sexual dysfunction in both men and women is chronic kidney disease. End-stage renal disease (ESRD) is a devastating condition that significantly impairs patients’ quality of life and length of life. Long-term hemodialysis or peritoneal dialysis, which can result in several physiologic and psychological changes, is part of the treatment for ESRD. As a result, sexual health is negatively impacted by ESRD [3–5].

**Chronic kidney disease (CKD) and hemodialysis can affect sexual function through hormonal dysregulation, vascular changes, and dialysis-related factors.** Hormonal imbalances like hypogonadism, hyperprolactinemia, and thyroid dysfunction can contribute to low libido, erectile dysfunction, and vaginal dryness. CKD accelerates atherosclerosis and neuropathy, compromising blood flow to the genitals and hindering sexual arousal and response. Additionally, rapid drops in blood pressure, excessive fluid removal, and potentially higher dialysis doses during hemodialysis treatment may further impact sexual function [6].

Few studies are reported from Sub-Saharan African Countries (SSA) regarding female sexual dysfunction (FSD) in (HD) patients. However, specific data on the prevalence of sexual dysfunction in this population in Somalia is unknown. To address this knowledge gap, the study aimed to determine the prevalence of sexual dysfunction and identify the associated factors among female hemodialysis patients in Somalia. The study involved a sample of female hemodialysis patients in Somalia who were assessed for sexual dysfunction using standardized measures.

## Method

Over a one-month period, a cross-sectional study was conducted among women with end-stage renal disease aged 18–50 years who were undergoing a dialysis program for at least three months at the dialysis centre of our institution. Those participants who were married, and were living with their partners were included in the study. Acute kidney injury requiring emergency dialysis, those with chronic kidney undergoing dialysis less than three months, older females (i.e., > 50 years), and divorced females or those not living with their partners were excluded from the study. Data regarding the sociodemographic features, clinical characteristics, frequency of sexual intercourse per week, and the Female Sexual Function Index (FSFI) scores were collected using a standard face-to-face interview questionnaire. The observational interview questionnaire was run by a gynaecologist in a suitable private setting and ensured patients’ privacy during the interview and the confidentiality of data.

The FSFI is a validated tool consisting of 19 questions for six domains, and the total score ranges between 2 and 36. The questionnaire is a patient-reported outcome measure assessing sexual function, and the scores are determined based on patient responses covering the last four weeks. The six domains are desire for two questions, arousal for four questions, lubrication for four questions, orgasm for three questions, satisfaction for three questions, and pain for three questions. An FSD is considered with a total score lower than 26.0 [4]. The questionnaire was not validated in the Somali language, and the authors used the original English version run by a gynaecologist experienced in the research field. Likert scale was used. All methods were carried out according to relevant guidelines and regulations.

The ethical approval form was reviewed by the ethical review board of Mogadishu Somalia Turkish Training and Research Hospital (REF No.10,271). All methods were carried out in accordance with relevant guidelines and regulations. Because of the sensitivity of the topic, the purpose of the topic was explained clearly to all of the participants, and all the study participants gave their informed consent for the use of their medical data for this research.

All statistical analyses were performed using the Statistical Package for Social Sciences (SPSS-IBM) for Windows Version 23. The frequencies and proportions were presented as point estimates in categorical variables and the mean (± SD) in quantitative variables. Student T-test was used to detect the significant association between the variables.

## Results

During the study period, a total of 115 participants were eligible for the study’s inclusion criteria. Overall, 152 female patients were undergoing a dialysis program; seven participants were older than 50, 13 were experiencing a dialysis program less than three months, six were divorced, and eleven did not live with their partners. The mean patient age was 38.5 ± 9.3 years (Table 1). The most common cause of ESRD was diabetes, which accounted for 53%, followed by hypertension (26.1%) and glomerulonephritis (9.6%). The mean duration of dialysis was 2.9 ± 1.4 years, and approximately two-thirds of the participants (62.5%) were in the program for more than three years. Regarding the frequency of sexual intercourse, 61.7% of female participants performed sexual intercourse less than once time/a week.

**Table 1:** Sociodemographic and clinical characteristics of the patients

Variable	No. of patients, Percentage %
Age	
Mean ±SD	38.5±9.3 years
18-35y	44 (38.3%)
36-50y	71 (61.7%)
**Duration of dialysis**	
Mean ±SD	2.97±1. 45 years
≤1yr	21(18.3%)
2-4yrs	22(19.1%)
≥4yrs	71(62.6%)
**Frequency of sexual intercourse per week**	
≤1 2	71(61.7%) 20(17.4%)
>2	24(20.9%)
**Causes of renal failure**	
Diabetes	61(53%)
Hypertension	30(26.1%)
Glomerulonephritis	11(9.6%)
Postrenal obstruction	3(2.6%)
Unknown	9(7.8%)
Polycystic kidney disease	1(0.9%)

The prevalence of FSD was 92.2% (*n* = 106) of all participants. The mean FSFI score of the participants was 16.05 ± 4.48. The distribution and mean scores of each domain are displayed in Table 2. The most affected domains in all female hemodialysis patients were sexual arousal and sexual orgasm. Most of the respondents revealed some degree of pain during sexual intercourse. Longer duration of dialysis program (i.e., more than four years), increasing age (i.e., > 35 years), those with diabetes had scored lower overall FSFI scores.

**Table 2:** Distribution of FSFI scores in each domain and overall score

Desire	2.66 ± 0.83
Arousal	2.71 ± 0.97
Lubrication	2.82 ± 1.16
Orgasm	2.71 ± 1.17
Satisfaction	2.82 ± 1.09
Pain	2.52 ± 0.76
Overall score	16.05 ± 4.48

## Discussion

Long-term renal replacement therapy patients, including those receiving hemodialysis and peritoneal dialysis, are subject to ongoing invasive procedures and medication therapy. Both male and female ESRD patients’ sexual health is significantly impacted [7]. This cross-sectional study investigated the prevalence and associated factors of sexual dysfunction in female hemodialysis patients in Somalia. The study found that the prevalence of sexual dysfunction in this population was 92.2%, which is significantly higher than the prevalence of sexual dysfunction in the general female population.

However, there is little evidence about risk factors for sexual dysfunction in haemodialysis women in the literature. Age, low educational level, depression, uraemia, history of sexual assault or STDs, and physical health condition have all been identified as risk factors for sexual dysfunction in women in published studies [8]. Age is the most significant risk factor for female sexual dysfunction [2].

In our study, the mean FSFI score of the participants was 16.05 ± 4.48, which is lower than the score of “post-menopausal women with sexual dysfunction” in the study of Kaplan et al. [9]. This indicates a high prevalence of sexual dysfunction among our hemodialysis patients.

In terms of associated factors, this study identified several variables that were significantly associated with sexual dysfunction in female haemodialysis patients. Age was found to be a significant factor, with older patients being more likely to experience sexual dysfunction. This finding is consistent with previous research indicating that older age is associated with increased prevalence of sexual dysfunction [10]. The association between advancing age and sexual dysfunction has previously been demonstrated in nonuremic individuals [11].

Diabetes is a commonly occurring comorbidity in hemodialysis patients, and it has been extensively associated with sexual dysfunction. For female hemodialysis patients, diabetes can contribute to sexual dysfunction through various mechanisms such as vascular complications, hormonal imbalances, and neuropathy [12]. Our study also aligns with this study, showing a significant association with diabetes patients.

There are several potential explanations for the relationship between the duration of dialysis treatment and sexual dysfunction. First, the physiological effects of chronic kidney disease (CKD) and hemodialysis treatment may contribute to sexual problems. Hemodialysis can lead to hormonal imbalances, nutrient deficiencies, anaemia, and electrolyte imbalances, which can all impact sexual health. Moreover, the longer a patient is on dialysis, the higher the cumulative exposure to these physiological factors, which could potentially worsen sexual dysfunction [13]. Psychological factors may also play a role in the association between longer duration of dialysis treatment and sexual dysfunction. Living with a chronic illness and undergoing regular dialysis sessions can lead to increased psychological distress, including anxiety, depression, and body image concerns. These psychological factors can negatively affect sexual desire, satisfaction, and overall sexual function [14].

ESRD’s impact on female sexual function plays a complex role with its underlying cause and concurrent conditions. Chronic diseases like diabetes can fuel its fiery steps, while cardiovascular problems and malnutrition become unwanted partners. ESRD’s own influence weakens hormonal rhythms and vascular flow, hindering arousal and pleasure. Addressing the root cause, managing co-occurring conditions, tailoring dialysis, and fostering open communication are key to restoring harmony and improving sexual well-being [15].

The study has several limitations, including, first, it is a single-centre study with a small sample size despite being the sole dialysis centre in Somalia. The study did not assess the anxiety and depression status of the patient, which can negatively correlate with FSD. Further studies are needed to examine the possible physiological changes that may lead to sexual dysfunction in hemodialysis patients, including Intradialytic changes in blood pressure, Ultrafiltration volume, and perhaps the dose of hemodialysis measured with Kt/V or URR. Despite these limitations, this is the first study that addresses this knowledge gap and aims to determine the prevalence of sexual dysfunction and identify the associated factors among female hemodialysis patients in Somalia.

## Conclusion

This study provides an initial report on the prevalence and associated factors of sexual dysfunction among female hemodialysis patients in Somalia. The high prevalence of sexual dysfunction emphasizes the importance of addressing sexual health concerns in this population. Healthcare providers should be proactive in assessing and managing sexual dysfunction in female hemodialysis patients by considering associated factors, such as age, duration of hemodialysis treatment, and comorbidities, to optimize patient care and improve quality of life.

## Data Availability

All study data and materials can be obtained from the corresponding author.
